# Detecting of the Longitudinal Grouting Quality in Prestressed Curved Tendon Duct Using Piezoceramic Transducers

**DOI:** 10.3390/s20041212

**Published:** 2020-02-22

**Authors:** Tianyong Jiang, Bin He, Yaowen Zhang, Lei Wang

**Affiliations:** 1 School of Civil Engineering, Changsha University of Science and Technology, Changsha 410114, China; tianyongjiang@csust.edu.cn (T.J.); hebinyeah@163.com (B.H.); 2 China Railway Fifth Survey and Design Institute Group CO., LTD, Beijing 102600, China; zywcsust@163.com

**Keywords:** piezoceramic transducer, prestressed curved tendon duct, longitudinal grouting quality, structural health monitoring

## Abstract

To understand the characteristics of longitudinal grouting quality, this paper developed a stress wave-based active sensing method using piezoceramic transducers to detect longitudinal grouting quality of the prestressed curved tendon ducts. There were four lead zirconate titanate (PZT) transducers installed in the same longitudinal plane. One of them, mounted on the bottom of the curved tendon duct, was called as an actuator for generating stress waves. The other three, pasted on the top of the curved tendon duct, were called as sensors for detecting the wave responses. The experimental process was divided into five states during the grouting, which included 0%, 50%, 75%, 90%, and 100% grouting. The voltage signals, power spectral density (PSD) energy and wavelet packet energy were adopted in this research. Experimental results showed that all the amplitudes of the above analysis indicators were small before the grouting reached 90%. Only when the grouting degree reached the 100% grouting, these parameters increased significantly. The results of different longitudinal PZT sensors were mainly determined by the distance from the generator, the position of grouting holes, and the fluidity of grouting materials. These results showed the longitudinal grouting quality can be effectively evaluated by analyzing the difference between the signals received by the PZT transducers in the curved tendon duct. The devised method has certain application value in detecting the longitudinal grouting quality of prestressed curved tendon duct.

## 1. Introduction

In recent decades, a large number of prestressed concrete long-span bridge have been built [1]. To improve the performance of the prestressed concrete bridges, the prestress is directly applied in concrete bridges through the post-tensioning method, which does not need the fixed tensioning equipment and the special construction site. However, grouting is one of the key processes in the construction of post-tensioning prestressed concrete bridges. Improper grouting leads to the invasion of moisture and, through chloride diffusion [2], causes the corrosion of steel reinforcement [3,4]. Grouting quality is one of the focus issues [5]. In the actual cement grouting, owing to the stoppage of the prestressed tendon duct, incorrect grouting method, poor expansion rate of cement slurry and other problems, it is inevitable that the grouting is not compacted and even the void appears in the prestressed tendon duct [6,7]. A prestressed tendon duct is generally embedded in the concrete bridges, and, with the characteristics of invisibility, it is difficult or impossible to directly observe the grouting quality. Therefore, it is of great importance to apply the non-destructive testing method to investigate the grouting quality of prestressed curved tendon duct [8,9].

Non-destructive testing (NDT) methods are widely used in practical engineering to detect damage degree of the civil engineering structures. NDT is to evaluate the health state of the structures and to determine whether the structure meets the actual requirements through the test results obtained non-destructively [10,11,12]. In practical engineering, the commonly used methods for monitoring grouting quality of prestressed tendon ducts include ground penetrating radar (GPR), core sampling, ultrasonic, and impact echo (IE), among others [13]. GPR has been used in civil engineering for decades, and the method itself has undergone considerable development, as has the measuring equipment [14]. Liu et al. inspected the ducts grouting defects by adopting the GPR method, and founded that radar could only monitor the first layer interface, but could not directly see more detailed media in the prestressed tendon duct [15]. Metallic media can easily reflect the radar impulse, making the GPR method unsuitable for ducts containing metallic materials, but it can be successfully applied for plastic ducts [16,17]. Core sampling detects internal defects by drilling samples in different parts of the structures, but it will cause some damage for the measured structures, and the slow monitoring speed makes it unsuitable for large-area monitoring [18]. Lu et al. made an investigation for the effects of various parameters on the application of ultrasonic in grouting quality, and found that when the velocity difference between concrete and cement slurry is small and the used reinforcement is a steel bar, ultrasonic can be used to inspect the grouting defect of post-tensioning prestressed tendon duct [19]. Han et al. used the ultrasonic impact-echo approach to evaluate the grouting quality and established certain numerical models to simulate the results with the finite element method. The experimental results are basically the same as the simulation predictions [20]. Terzioglu et al. utilized ultrasonic to inspect the defect location and severity in a full-size, post-tensioned U-girder specimen. The study clearly showed that in the anchorage area, ultrasound can estimate the severity of the grouting defect in the internal tendons [21]. As a non-destructive testing method with single detection surface, the impact-echo method is widely used in concrete structures [22]. Zou et al. utilized the impact-echo approach to carry out construction field test to use various grouting stages in post-tensioned box girders. The coordinates are obtained by mathematical analysis of the main frequency of each test point, and the contour image is drawn. Through the comparison of the contour image and actual situation, the grouting defects of the ducts can be verified certainly [23]. Fursa et al. presented results of studying the spatial variation of parameters of the electrical response to a weak impact under stepwise loading of concrete beams reinforced with steel rods. The results showed that impact-echo can be used to locate the defective area in reinforced concrete under bending conditions [24]. However, the IE has prominent limitations. The low frequency stress wave leads to low monitoring accuracy and it is unable to effectively monitor large structures [25,26,27]. These nondestructive testing technologies have their own shortcomings in detecting the grouting quality of prestressed tendon ducts. Therefore, an inexpensive, timely monitoring method to overcome these shortcomings is needed to detect the grouting defect of prestressed ducts.

In recent years, piezoceramics transducers have been used to monitor structural health [28,29,30]. Piezoceramic transducers have their superiorities of wide frequency range [31,32,33], low cost [34,35], energy harvesting [36,37,38], and the dual functions as a sensor and an actuator. Among various piezoceramic materials, lead zirconate titanate (PZT) has the strongest piezoelectric effect and can be fabricated in different shapes [39,40]. With proper protection, various PZT based transducers that are suitable for deployment in civil structures, including the concrete structures, have been developed [41,42,43,44], and have found application in bridge structural health monitoring [45,46,47]. The active sensing method based on piezoceramic transducers is to bond PZT transducers on the surface of structure [48] or embed piezoelectric transducers in the structure [49], and then the actuator generates the signals, which is detected by the sensors. Finally, the damage state is monitored and evaluated according to the stress wave signal [50,51]. Song et al. detected the crack propagation inside the structure by embedding PZT sensors into reinforced concrete beams. The experimental results show that piezoelectric transducers can predict the failure of concrete structures [52]. Du et al. used the active sensing method based on piezoelectric transducers to monitor the severity of duct cracks and established the crack severity index based on wavelet packet to quantitatively identify the damage of ducts at different cracks [53]. Hu et al. deduced the elastic solution of wave propagation generated by PZT and obtained the viscoelastic solution from the elastic solution through mathematical analysis. Then according to the interaction effect of PZT structure, the output voltage of PZT sensor is calculated [54].

PZT based methods have also been reported in grout quality monitoring. Jiang et al. used PZT transducers to detect grouting quality of the prestressed ducts based on an active sensing approach. In this study, the signals received by PZT sensor was calculated by wavelet packet-based energy analysis. Experimental investigation shows that the grouting quality of the ducts can be reflected by the energy level of the PZT sensors [55]. Tian et al. applied time-reversal technique to detect grouting defects using piezoelectric transducers. Experimental investigation shows that the grouting defects can be got by analyzing the peaks in the data [56]. Luo et al. used ultrasonic time-of-flight (TOF) approach to monitor the filling conditions of concrete ducts filled with fiber-reinforced polymers and verified the feasibility of the TOF method to detect the filling conditions [57]. Jiang et al. used finite element analysis to establish a two-dimensional model to simulate the change of energy under different grouting stages in order to further study the propagation mode of stress waves in piezoelectric transducers [58].

At present, the commonly used data processing methods of structural health monitoring mainly include time-domain analysis, frequency-domain analysis, and wavelet packet-based energy method. The time-domain signals are the original responses collected by the sensors, which can be described as a curve of signals with time [59]. Frequency-domain signals, transformed by Fourier transform from time-domain signals, are used to calculate the power spectral density (PSD) energy, which are closely related to the frequency composition of the signals. In time-domain analysis of the signals, sometimes the time-domain parameters of some signals are the same, but it does not mean that the signals are exactly the same [60]. This is because the signals not only change with time, but also are related to frequency, phase and other information. Therefore, it is necessary to further analyze the frequency composition of the frequency-domain signals. As a result, in the signal processing, the frequency-domain analysis can obtain the sensitive frequency range and the corresponding PSD energy on detecting the grouting compactness of the prestressed tendon duct. In addition, wavelet packet decomposition can be performed on the signal sequence during the signal processing, and then the wavelet packet energy spectrum of the signals can be extracted. The study results show that the sensitivity and stability of the eigenvalues composed of wavelet packet energy spectrum are better than those in time-domain analysis and frequency-domain analysis [59,61]. Therefore, the above three signal analysis methods have their own focuses in the data process. The time-domain analysis reflects the strength of the original signals. The frequency-domain analysis reflects the sensitivity of each decomposition frequency of the structure, and the wavelet packet energy can better identify the small grouting defects.

The literature review shows that most of the studies on grouting quality are on the cross section of prestressed duct. However, there are few studies along the longitudinal section of prestressed curved tendon duct. In actual prestressed concrete bridge engineering, the prestressed tendon ducts are curved along the length direction of the bridge, and the grouting quality in longitudinal positions is different in general. Therefore, this paper applies a stress wave-based active sensing method using piezoceramic transducers to detect the longitudinal grouting quality of the prestressed curved tendon duct in real time. In this experiment, one PZT transducer regarded as an actuator was installed on the bottom of the duct and was applied to generate the stress wave signals. Three PZT transducers regarded as sensors and installed on the top of the curved tendon duct were used to detect the above signals. The experimental process was divided into five states during the grouting, which included 0%, 50%, 75%, 90%, and 100% grouting. The sensors at the top of the curved tendon duct received directly the signal only when the tendon duct was fully filled with grouting materials. The voltage signals of time-domain, PSD energy of frequency-domain and wavelet packet energy were adopted in a follow-up analysis of this research.

## 2. Detection Principle

### 2.1. Piezoelectric Sensing Method

**Figure 1:**
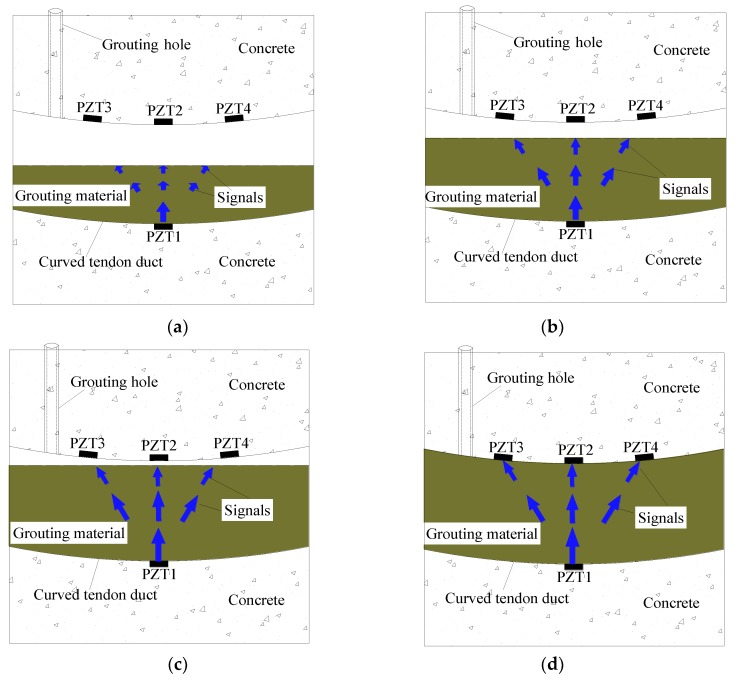
Schematic diagram of detecting the longitudinal grouting quality. (**a**) 50% grouting; (**b**) 75% grouting; (**c**) 90% grouting; (**d**) 100% grouting.

In this study, a stress wave-based active sensing method using piezoceramic transducers was employed to monitor the longitudinal grouting quality of prestressed curved tendon ducts. In this experiment, the grouting quality of prestressed curved tendon ducts along the longitudinal direction will be studied. The schematic diagram of detecting the longitudinal grouting quality of prestressed curved tendon ducts was given in Figure 1. The piezoceramic transducers were pasted on the lower and upper surface of the curved tendon duct. Among them, the PZT 1 regarded as an actuator was arranged at the lower part of the curved tendon duct; the PZT 2, 3, 4 regarded as sensors were pasted on the upper part of the curved tendon duct for receiving stress wave signals.

The grouting material as a bond medium, injected into the prestressed curved tendon duct through the grouting hole, can be used to transmit the stress wave signals. When there was no grouting in the prestressed curved tendon duct, the sensors cannot directly receive the signals generated by the actuator. The sensors PZT 2, 3, 4 can only receive very small and negligible signals transmitted through the prestressed curved tendon duct wall. When grouting quality of the prestressed curved tendon duct reaches 50%, 75%, and 90%, respectively, although the signals of PZT 2, 3, 4 sensors are enhanced, their amplitudes are still small, which is mainly caused by the fact that the prestressed curved tendon duct still does not reach full quality at these states and the signals cannot be directly transmit to the sensors, as shown in Figure 1a–c. Where, in the above grouting state, if the curved duct is completely filled with grouting material, the state of complete compaction is called 100% grouting. If the curved duct is empty, it is called 0% grouting. The remaining states are respectively the mass percentage of the grouting material in the current state and the grouting material in the fully compacted state.

When the curved tendon duct reaches 100% grouting, the signals can be received by the sensors through the grouting material, as shown in Figure 1d. PZT 2, 3, 4 sensors can receive strong signals generated by the actuator PZT 1. Moreover, due to the characteristic of curve of the prestressed curved tendon duct, PZT 2 sensor located in the nearest position to PZT 1 actuator first receives the full signals when the curved tendon duct is fully compacted along the transmission direction between the sensors and actuator. Subsequently, PZT 3, 4 sensors gradually receive sufficient large signals as the longitudinal quality of the curved tendon duct increases.

### 2.2. Wavelet Packet-Based Energy Method

As is known to all, to detect grouting quality of prestressed tendon duct, the key is to effectively analyze and identify the signals received by the sensors. Wavelet packet energy analysis has better advantages in the sensitivity and stability of the eigenvalues than time-domain and frequency-domain analysis. Past researches have shown that the wavelet package-based energy is sensitive to cracks in concrete structures [62,63]. When the stress wave propagates inside the prestressed curved tendon duct, due to the duct grouting defects, reflection attenuation [64,65], scattering attenuation, and absorption attenuation of stress wave will result in a large loss of stress wave energy. Therefore, the defect degree of the curved tendon duct can be judged according to the change of energy. In recent years, researchers have studied the signal analysis and signal data processing progress by utilizing the wavelet packet-based energy method.

In the energy analysis based on wavelet packet, the received signal $X_{i}$ can be decomposed into 2^n^ frequency bands by an n-level wavelet packet. Then the decomposed signal $X_{i,j}$ can be expressed as

(1) $$X_{i,j}=\left[X_{i,j,1},X_{i,j,2},\cdots ,X_{i,j,m}\right]$$

where, m is the number of sampling data, i stands for the various time and j is the corresponding frequency band $\left(j=1,2,3,\cdots ,2^{n}\right)$. The energy $E_{i,j}$ of the decomposed signal $X_{i,j}$ can be expressed as

(2) $$E_{i,j}={\left\|X_{i,j}\right\|}^{2}={X_{i,j,1}}^{2}+{X_{i,j,2}}^{2}+\cdots +{X_{i,j,m}}^{2}$$

The signal energy $E_{i}$ of the sensor signal $X_{i}$ at different time can be given as

(3) $$E_{i}=\left[\begin{array}{ccccc} E_{i,1}, & E_{i,2}, & E_{i,\;3}, & \cdots & E_{i,2^{n}} \end{array}\right]$$

During detecting grouting quality of prestressed curved tendon duct, the sensor signals are collected in the grouting order. Firstly, the initially collected signal energy is $E_{0}=\left[\begin{array}{ccccc} E_{0,1}, & E_{0,2}, & E_{0,\;3}, & \cdots & E_{0,2^{n}} \end{array}\right]$ at the 0% grouting state when the prestressed curved tendon duct is empty; then, collected signal energy is regarded as $E_{i}=\left[\begin{array}{ccccc} E_{i,1}, & E_{i,2}, & E_{i,\;3}, & \cdots & E_{i,2^{n}} \end{array}\right]$ according to different grouting states. When the curved duct is completely filled, the signal energy $E_{full}$ is collected. With the increasing grouting quality, the signal energy $E_{i}$ will increase, and it will reach to its maximum when $E_{i}$ gets to $E_{full}$.

## 3. Experimental Equipment and Process

### 3.1. Specimen Component

**Figure 2:**
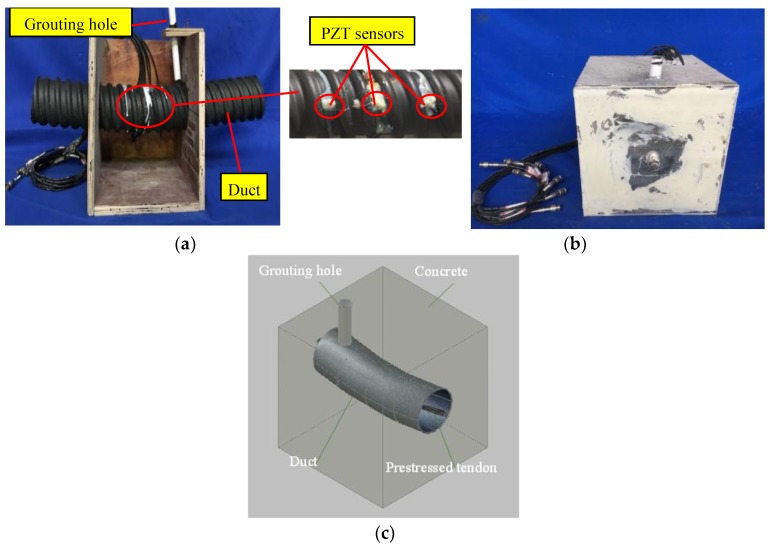
The test specimen. (**a**) The frame work before pouring the concrete. (**b**) The test specimen after the concrete is poured. (**c**) Three-dimensional view of the model.

In order to investigate the longitudinal characteristics of grouting quality of prestressed curved tendon duct, an active sensing approach based on piezoceramic transducers was used. Based on the actual engineering practice, the prestressed curved tendon duct was designed with a certain degree of downward curvature in this research. Figure 2 shows the frame work before pouring the concrete, the test specimen after the concrete is poured, and three-dimensional view of the model.

**Figure 3:**
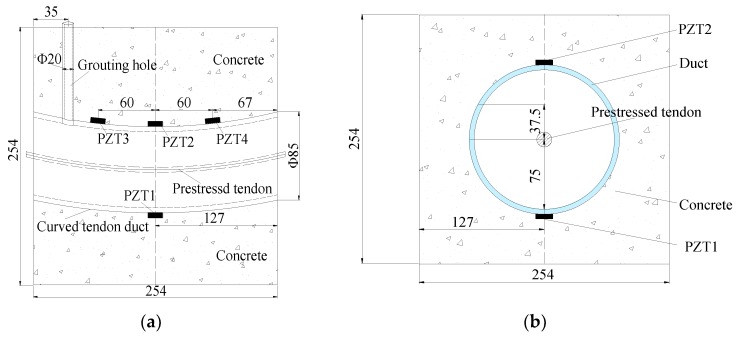
Specimen dimensions (unit: mm). (**a**) Longitudinal section. (**b**) Transverse section.

The test specimen mainly included the prestressed tendon, the plastic curved duct, the concrete, the grouting material, the PZT transducers, the lead wires and the BNC connectors. The concrete mixture included Portland cement PO 42.5, sands, gravel, and water. In order to improve the fullness and compactness of the prestressed curved tendon duct and enhance the fluidity of the grouting material, the grouting material adopted Portland cement PO 42.5 and the water-cement ratio of the pure cement slurry was 0.40. The grouting tube is PVC pipe. There were two transparent plastic plates bonded on the two sides of the test specimen to avoid the spillage of grouting material and ensure real-time detecting the quality level of the prestressed curved tendon duct. The specimen dimensions are shown in Figure 3. The specimen was a cube with a length of 254 mm on each side, the outer and inner diameters of plastic curved duct were 85 mm and 75 mm, respectively. Meanwhile, in order to facilitate the grouting of prestressed curved tendon duct, a PVC pipe with an inner diameter of 20 mm was adopted as the grouting pipe.

Four PZT transducers were bonded on the surface of the curved tendon duct, and the detailed layout is shown in Figure 3a. It can be seen that PZT 1, 2 were installed on the bottom and top of the duct center section, respectively; PZT 3, 4 were symmetrically arranged on the top of the duct with the distance of 60 mm from the center line of the test specimen. In addition, PZT 1 regarded as an actuator was used to generate the signals, and PZT 2, 3, 4 regarded as the sensors were utilized to receive the signals. The PZT-5H materials with the mode of d33 (compression type) and single-piece structure used in this research were suitable to cut into small patches as transducers. The PZT patches are 7.4 mm in diameter and 1.3 mm in thickness. The PZT parameters provided by the manufacturer in the test specimen are shown in Table 1.

### 3.2. Experimental Equipment

**Figure 4:**
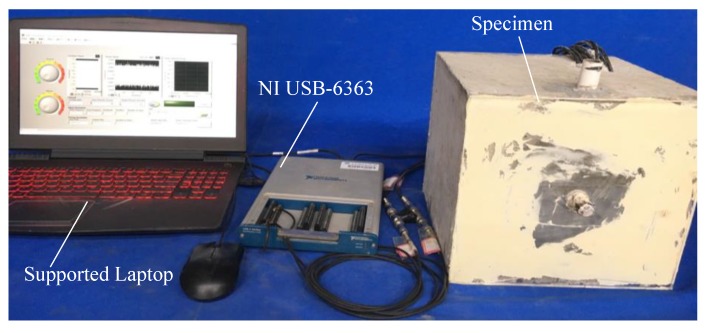
Experimental equipment.

The experimental equipment included a specimen, a supported laptop and a NI USB-6363, as shown in Figure 4. The data acquisition board NI USB-6363 was utilized to produce the signals to the PZT 1 actuator and collect the signals from the PZT 2, 3, and 4 sensors.

### 3.3. Experimental Process

**Figure 5:**
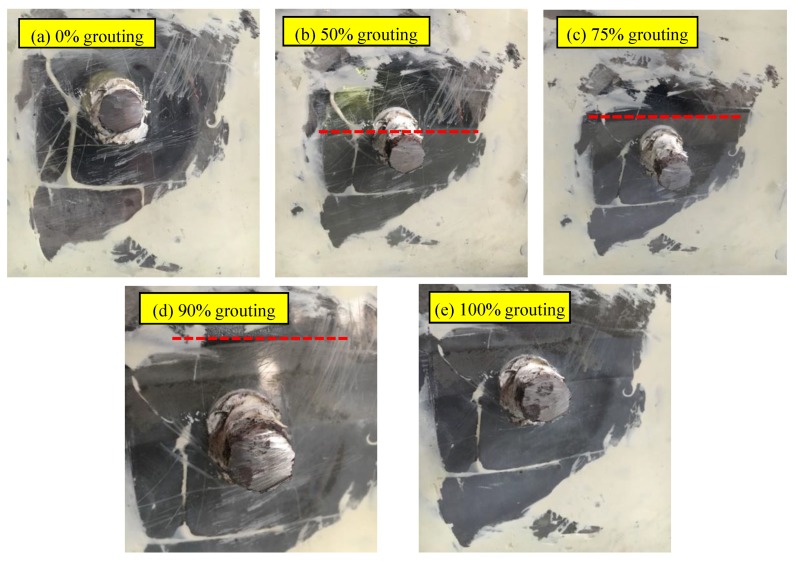
Test specimen in different states.

In practice, the grouting defect often occurs at the bending position of tendon ducts. Based on the actual engineering situation, this test designed five grouting conditions to simulate the different states, including 0%, 50%, 75%, 90%, and 100% grouting, as shown in Figure 5.

The grouting materials were carefully poured into the specimen through the grouting tube. The interval of each condition was 5 days. The grouting height in the curved duct can be seen through the transparent plastic plate and the grouting proportion can be controlled by measuring the grouting height. The distance between the level of half grouting and the bottom of test specimen is 127 mm. The distance between the level of 75% grouting and the bottom of test specimen is 148 mm. The distance between the level of 90% grouting and the bottom of test specimen is 161mm and the level of 100% grouting was 169.5 mm.

**Figure 6:**
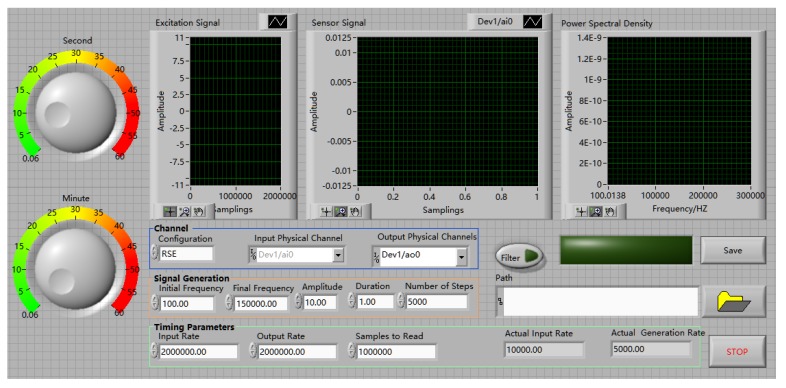
Operating interface of the special test program of NI USB-6363.

The data acquisition board NI USB-6363 was a 32 AI (16-Bit, 2 MS/s), 4 AO (2.86 MS/s), 48 DIO USB Multifunction I/O Device. When the NI USB-6363 was utilized to detect the test specimen, the actuator PZT 1 and the sensor PZT 2 or 3 or 4 were first connected to the device. Then opened the special test program, which was compiled by LabVIEW software. Its operating interface is shown in Figure 6. Next, selected the corresponding input and output channels on the program interface, and filled in a stable sweep signal as the excitation signal, including initial frequency 100 Hz, final frequency 150 kHz, amplitude 10 v, duration 1 s of one cycle, 5000 times of sweeps from initial frequency to final frequency, then determined input or output rate and the path to read the data. Finally, the program was executed to obtain the response signal of the test specimen, and the data was processed to evaluate the grouting quality of prestressed curved tendon duct.

In order to fully and deeply understand the reliability of the active sensing approach based on piezoceramic transducers, the time-domain analysis, frequency-domain analysis and wavelet packet-based energy method are proposed for the signals collected by PZT 2, 3, and 4 sensors under different grouting quality in the subsequent data analysis process. Among them, the time-domain analysis studies the variation trend of the voltage signals with grouting degree. The frequency-domain analysis mainly evaluates the frequency sensitive range and PSD energy of the structural responses, and the wavelet packet energy identifies the small changes in grouting quality.

## 4. Experimental Result and Analysis

### 4.1. Time-Domain Analysis

**Figure 7:**
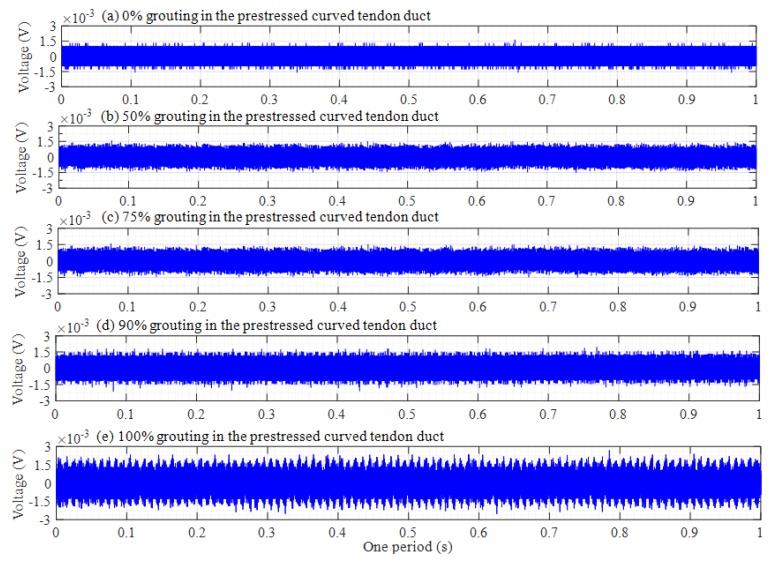
Voltage signals of PZT 2 in one period.

**Figure 8:**
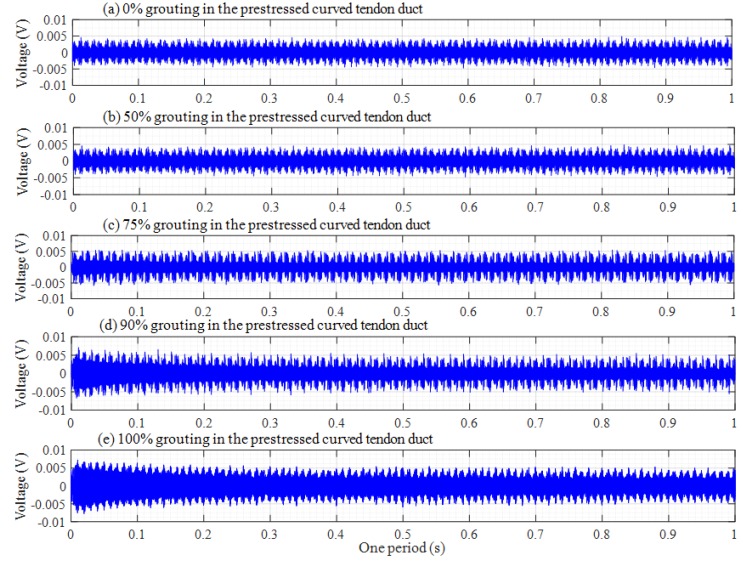
Voltage signals of PZT 3 in one period.

**Figure 9:**
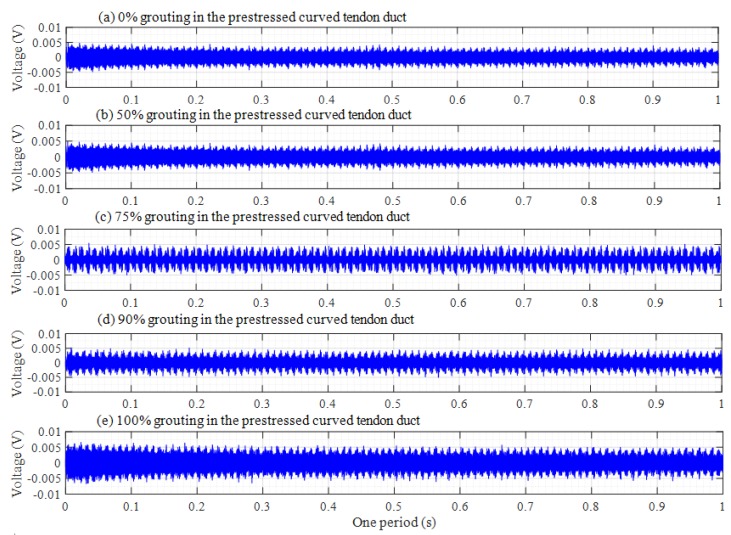
Voltage signals of PZT 4 in one period.

The original responses collected by the PZT sensors are time-domain signals. The attenuation trend of this amplitude will increase with the increasing grouting degree in time-domain analysis. Th voltage signals of PZT sensors in one period are shown in Figure 7, Figure 8 and Figure 9, among which, Figure 7, Figure 8 and Figure 9 respectively show the voltage signals of PZT 2, 3, and 4 sensors in different grouting states. Each plot represents the signal received by a sensor under five different grouting states, including 0%, 50%, 75%, 90%, and 100% grouting.

From Figure 7, it can be seen that signal voltage of PZT 2 pasted on top of center section of prestressed curved tendon duct increases with the increasing grouting quality. However, the increase of the signal voltage is very small, or almost zero between 0% and 75% grouting. The reason is that until the grouting degree reaches 75% grouting, stress waves cannot be transmitted directly through the grouting materials, but through the wall of prestressed curved tendon duct. Stress waves can firstly propagate a certain distance in the grouting medium, when the grouting level arrives at 90%, and then travel around the wall of prestressed curved tendon duct, which results in obvious increments for the signal amplitude. When prestressed curved tendon duct is fully compacted, the stress waves can be transmitted directly from the PZT 1 actuator to the PZT 2 sensor through the grouting medium, which leads to a large increase in stress wave amplitude.

Figure 8 shows the voltage signals of PZT 3. It is obvious that Figure 8 is different with Figure 7. If the grouting degree is less than 50%, the amplitude of the signal does not increase significantly. Subsequently, the amplitude of the signal will gradually increase with the grouting degree increasing. The signal amplitude reaches the maximum until the grouting is fully compacted. This is mainly because PZT 3 is the closest to the grouting hole, as the grouting process progresses gradually. The slurry of the tendon duct wall near PZT 3 sensor will also increase, which will lead to an increase in the amplitude of stress wave when the stress wave signals propagate through the wall of the tendon duct. For PZT 4 sensor in Figure 9, the distance from the grouting hole is farther than that of PZT 2 sensor, and the signal amplitude changes with the increasing grouting degree are almost similar to that of PZT 2 sensor, that is, the signal amplitude changes slightly before 90% grouting, and only the PZT 4 sensor can receive strong signals in the 100% grouting state.

From the above time domain diagram, it can also be seen that the voltage amplitude decreases gradually with time during one period, which is especially obvious in the 100% grouting state. As is known to all, the stress wave response with lower frequency attenuates much less than that with higher frequency. Since the excitation signal was a sweep sine wave from a low frequency 100 Hz to a high frequency 150 kHz with respect to time, it can be known that the amplitude of the signal received by the PZT sensor decreases with the increase of frequency.

### 4.2. Frequency-Domain Analysis

**Figure 10:**
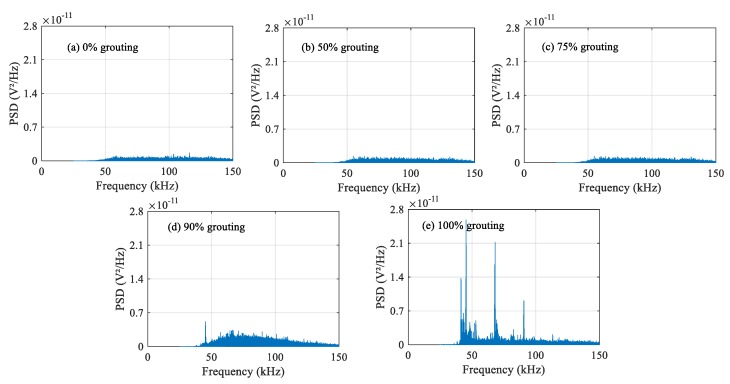
PSD energy of PZT 2 in different grouting states.

**Figure 11:**
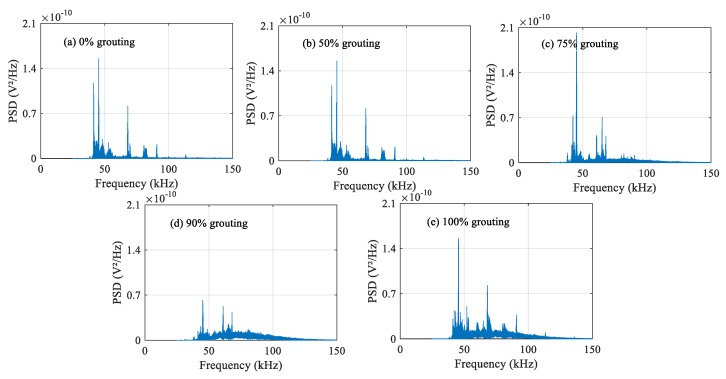
PSD energy of PZT 3 in different grouting states.

**Figure 12:**
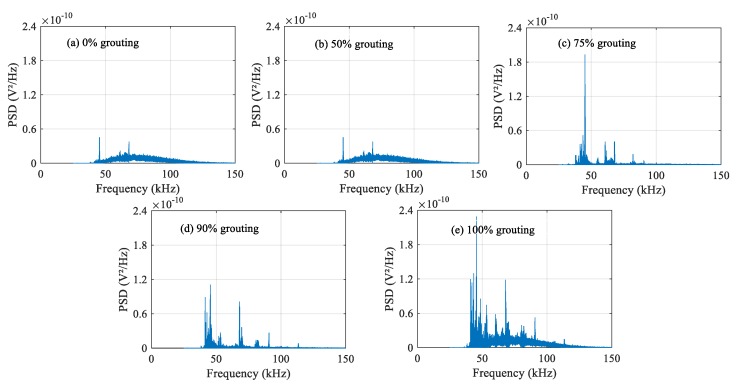
PSD energy of PZT 4 in different grouting states.

The damage level of structures can be determined by analyzing the attenuation degree of signals at different frequencies. The frequency-domain signals of each PZT sensor under different grouting states are shown in Figure 10, Figure 11 and Figure 12. Frequency-domain signals are transformed by Fourier transform from time-domain signals, so the power spectral density (PSD) energy is more obvious than the voltage signal received by the sensor. The PSD energy of PZT 2 in different grouting states is shown in Figure 10. The PSD energy of PZT 3 in different grouting states is shown in Figure 11. The PSD energy of PZT 4 in different grouting states is shown in Figure 12. Each plot represents the frequency-domain signal received by a sensor under five different grouting states, including 0%, 50%, 75%, 90%, and 100% grouting.

These frequency-domain signal diagrams indicate that the sensor signal has a sensitive frequency range from 50 kHz to 100 kHz, which is similar to the experimental findings in references [59,66]. The sensitive frequency range and PSD energy peak in the frequency-domain signals mainly depend on the characteristics of the structural responses. It can be seen in Figure 10 that the PSD energy of PZT 2 increases with the increasing grouting quality. However, the PSD energy increases very little from the case of 0% grouting to the case of 75% grouting. When the grouting state reaches 90%, the PSD energy increases obviously. This change is even more pronounced than the time-domain diagram. When the prestressed curved tendon duct is fully compacted, the stress waves can be transmitted directly from the PZT 1 actuator to the PZT 2 sensor through the grouting medium, which leads to a large increase in PSD energy. Compared with Figure 10, the PSD energy of Figure 11 and Figure 12 show the similar variation trend in the different states.

### 4.3. Wavelet Packet Energy

**Figure 13:**
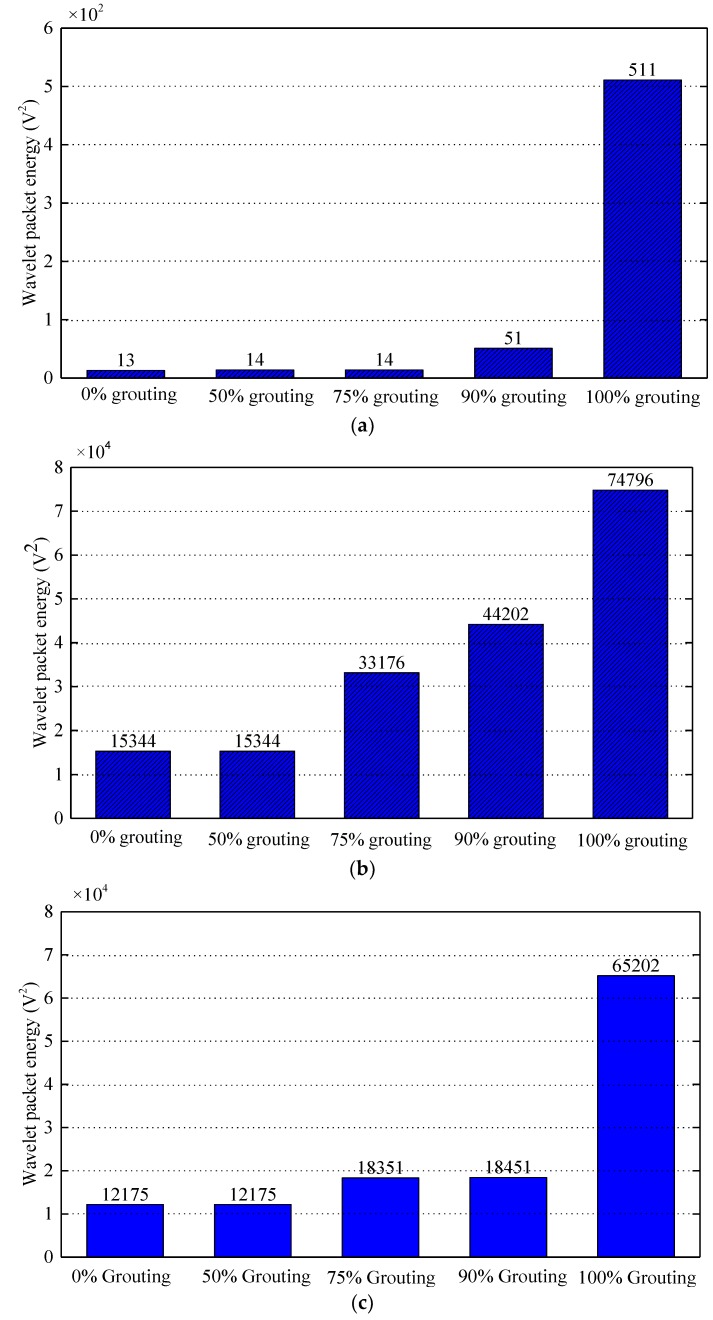
Wavelet packet energy of PZT sensors in different grouting states. (**a**) PZT 2 sensor; (**b**) PZT 3 sensor; (**c**) PZT 4 sensor.

According to previous wavelet packet energy, there is a close relationship between wavelet packet energy and grouting quality of prestressed curved tendon duct. The signals received by the sensors can be expressed quantitatively by wavelet packet energy. The wavelet packet energy of PZT sensors under different grouting states is shown in Figure 13. The wavelet packet energy of PZT 2, 3, and 4 in different states are shown in Figure 13a–c respectively.

It can be seen in Figure 13a that the wavelet packet energy of PZT 2 mounted on the top of center section of prestressed curved tendon duct almost does not increase with the growth of grouting quality from 0% grouting to 90% grouting. The reason is that before the grouting degree reaches 90%, the stress wave signals propagated via the wall of prestressed curved tendon duct. It will result in little change in wavelet packet energy. When it comes to 100% grouting, the wavelet packet energy of PZT 2 suddenly increases. It is mainly because during 100% grouting, the stress wave signals propagated via the grouting materials between actuator PZT 1 and sensor PZT 2.

Compared with Figure 13a, Figure 13b shows that the wavelet packet energy of PZT 3 does not increase from 0% grouting case to half grouting case, when the grouting state comes to 75% and the wavelet packet energy of PZT 3 is almost doubled. The wavelet packet energy of PZT 3 also increases with the growth of grouting quality in the following grouting states. The reason for the difference is that the PZT 3 is the closest to the grouting hole, as the grouting process progresses gradually, the slurry of the tendon duct wall near PZT 3 will also increases, which will lead to an increase of the wavelet packet energy when the signals propagate through the wall of the duct. For the wavelet packet energy of PZT 4 in Figure 13c, it shows the similar trend with PZT 2. The wavelet packet energy of PZT 4 does not increase between 0% grouting and half grouting. When it comes to the grouting state between 75% and 90%, the wavelet packet energy gradually increases. Until it comes to 100% grouting, the wavelet packet energy of PZT 4 increases significantly.

### 4.4. Analysis and Discussion

In summary, from the voltage signal, PSD energy and wavelet packet energy, it can be seen that their amplitudes are relatively small before the grouting degree reaches 90%, and the changes are relatively small with the increasing grouting degree. Only when the grouting degree reaches the full compactness, the amplitudes increase significantly. The amplitude difference along the longitudinal PZT sensors is mainly determined by the distance from the generator, the position of grouting holes, and the fluidity of grouting materials. Therefore, this paper not only verifies the conclusion in previous research for the transverse grouting quality, but also further develops the relationship between the grouting quality of prestressed curved tendon duct along the longitudinal direction and the propagation of stress wave.

In addition, it should be noted that since the proposed method is limited to detecting the local grouting quality of the prestressed curved tendon duct, there is still much work to be done for monitoring the grouting quality of the large-scale structures in the practical applications in the future, such as the optimal arrangement method of PZT transducers, calibration, normalization and standardization of signal processing, correlation analysis of input and output signals, and isolation and protection of the surface mounted PZT patches, especially in harsh environments.

## 5. Conclusions

This paper takes full advantages of piezoelectric materials in intelligent monitoring of structures and proposes a new method for detecting the longitudinal grouting quality of prestressed curved tendon duct based on active sensing method. The feasibility and effectiveness of the proposed method to detect the grouting quality of duct were verified by experiments. In this research, PZT 1 arranged at the bottom of duct was used to generate stress waves as an actuator. As sensors, PZT 2, 3, 4 were mounted on the top of the duct to receive the signals. The prestressed curved tendon duct was designed with a certain degree of downward curvature in this test specimen. The experimental process was divided into five stages during the grouting, which included 0%, 50%, 75%, 90%, and 100% grouting. Only when the grouting was full, the sensors could directly receive the signals. By comparing the voltage signals, PSD energy and wavelet packet energy of the piezoelectric sensors under different grouting states, the grouting level of the prestressed curved tendon duct can be estimated. In conclusion, the study results of detecting the longitudinal grouting quality show that the test results are closely related to the longitudinal boundary of the prestressed curved tendon duct, such as PZT transducers distribution, grouting hole position, and fluidity of grouting material.

## Figures and Tables

**Table 1:** PZT parameters provided by the manufacturer in the test specimen.

Poisson Ratio (*v*)	Piezoelectric Constant *d*_33_ (pC/N)	Dielectric Constant *ε* (F/m)	Dielectric Loss *tgδ*	Electromechanical Coupling Coefficient (*k*)	Curie Temperature T*_c_* (°C)	Density *ρ* (g/cm^3^)
0.34	640	3400	1.3	0.68	250	7.6
